# A randomised controlled trial of ‘clockwise’ ultrasound for low back pain

**DOI:** 10.4102/sajp.v72i1.306

**Published:** 2016-07-28

**Authors:** Adriaan Louw, Kory Zimney, Merrill R. Landers, Mark Luttrell, Bob Clair, Joshua Mills

**Affiliations:** 1 International Spine & Pain Institute, United States; 2 Department of Physical Therapy, School of Allied Health Sciences, University of Nevada Las Vegas, United States; 3 Department of Physical Therapy, St. Ambrose University, United States; 4 Department of Physical Therapy, School of Health Sciences, University of South Dakota, United States; 5 Bird Physical Therapy, United States; 6 Clair Physical Therapy, United States; 7 Wasatch Peak Physical Therapy, United States

## Abstract

**Aims:**

To examine how the choice of words explaining ultrasound (US) may influence the outcome of physiotherapy treatment for low back pain (LBP).

**Methods:**

Sixty-seven patients with LBP < 3 months were randomly allocated to one of three groups – traditional education about US (control group [CG]), inflated education about US (experimental group [EG]) or extra-inflated education about US (extra-experimental group [EEG]). Each patient received the exact same application of US that has shown clinical efficacy for LBP (1.5 Watts/cm^2^ for 10 minutes at 1 Megahertz, pulsed 20% over a 20 cm^2^ area), but received different explanations (CG, EG or EEG). Before and immediately after US, measurements of LBP and leg pain (numeric rating scale), lumbar flexion (distance to floor) and straight leg raise (SLR) (inclinometer) were taken. Statistical analysis consisted of mixed-factorial analyses of variance and chi-square analyses to measure differences between the three groups, as well as meeting or exceeding minimal detectable changes (MDCs) for pain, lumbar flexion and SLR.

**Results:**

Both EG and EEG groups showed a statistically significant improvement for SLR (*p* < 0.0001), while the CG did not. The EEG group participants were 4.4 times (95% confidence interval: 1.1 to 17.5) more likely to improve beyond the MDC than the CG. No significant differences were found between the groups for LBP, leg pain or lumbar flexion.

**Conclusion:**

The choice of words when applying a treatment in physiotherapy can alter the efficacy of the treatment.

## Introduction

In research design where scientists aim to examine the efficacy of a treatment modality, it is common practice to eliminate as many variables as possible to ‘only’ assess the effect of the treatment modality (Moseley & Mead 2004). In physiotherapy, for example, following the execution of the trial, a study often reports on the efficacy of such an intervention in regards to changes in pain, range of motion, function and psychometric properties (Bogduk 2007). Even with stringent research design, such as inclusion and exclusion criteria, or even trying to replicate consistency in delivery of a treatment, there are various innate issues surrounding the experiments that may or may not influence the outcome of a research project (Moseley & Mead 2004). These issues are often referred to as non-specific effects.

In recent years, especially with the interest in pain neuroscience, attention has shifted to therapeutic alliance (TA) (Fuentes *et al*. 2014; Joyce *et al*. 2003). TA is defined as the working rapport or positive social connection between the patient and the therapist (Crepeau & Garren 2011). By virtue of its definition, TA is a complex blend of therapist technical skill, verbal and non-verbal communication, sense of warmth, trust and collaboration (Crepeau & Garren 2011). Aside from TA, various clinical factors of the environment, such as colours, smells and sounds, also influence the outcome of a proposed treatment (Street, Gordon & Haidet 2007). One factor, heavily associated with TA, is the words the physiotherapist chooses in explaining a test or treatment. It is well established that word choice by healthcare providers can have a positive or negative influence on their patients. For example, Coppieters *et al*., while performing a straight leg raise (SLR) on patients with lumbar radiculopathy, altered their words to describe the test as either a test of ‘muscle’ or ‘nerve’, resulting in a significantly reduced SLR in patients who believed the test was a test of ‘nerve’ versus ‘muscle’ (2005). The researchers proposed that the word ‘nerve’ was more associated with pain than ‘muscle’ and thus resulted in the substantial difference. In a psychology study, hotel workers were informed that their daily work of cleaning rooms was actually good for them and met the Surgeon General’s recommendation for daily physical exercise (Crum & Langer 2007). After 4 weeks, compared to a control group (CG) who did not receive such information, the informed workers perceived themselves to be getting significantly more exercise, which resulted in significant differences in weight, blood pressure, body fat and waist-to-hip ratio (Crum & Langer 2007). Conversely, it has been shown that words may also induce more fear, limit movement and increase a pain experience (Ott *et al*. 2012; Wilson, Williams & Butler 2009). Nursing studies examining the effect of words during injections have shown that the word ‘sting’ compared to ‘beware’ prior to the needle-prick resulted in a significant increase in pain experience (Ott *et al*. 2012). Sloan *et al*. showed that patho-anatomical words associated with back pain, such as ‘wear and tear, deterioration, disc space loss, crumbling’ and ‘collapsing’ are associated with increased fear and anxiety (Sloan & Walsh 2010). What words scientists, and ultimately clinicians, chose to explain a test or procedure does matter. Words may enhance or hurt the therapeutic effect of a proposed treatment modality. In fact, it is also proposed that the explanation of a treatment may be more beneficial than the actual treatment technique. The aim of this study was to examine how the choice of words explaining a treatment may influence the outcome of a physiotherapy treatment.

## Methods

### Study Design

**FIGURE 1:**
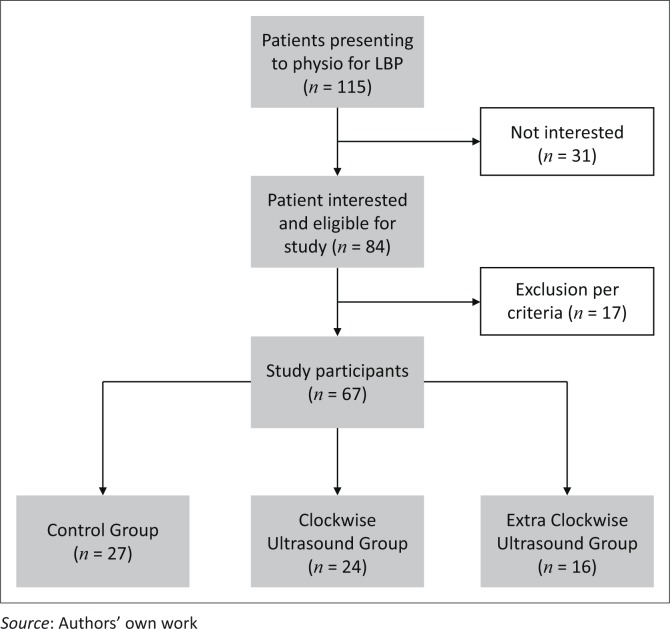
Study layout.

Ethics approval was obtained by the Internal Review Board (IRB) of The University of South Dakota. This was a randomised controlled trial where participants were randomly assigned to either one of two experimental groups (EG; extra-experimental group [EEG]) or CG (Figure 1). All patients acknowledged their understanding and willingness to participate by providing signed consent. Participants were informed that the study was aimed at determining the efficacy of therapeutic ultrasound (US) on people with low back pain (LBP).

### Randomisation

Randomisation was performed, using an alternating envelope system. Upon presenting at the clinical site, physiotherapists drew an envelope, which randomly assigned the patient to EG, EEG or CG. The envelopes contained identical information, except that description of the US was different for the EG, EEG and CG.

### Setting

An educational seminar company specialising in pain science for physiotherapists posted an advertisement regarding the study in its newsletter. Physiotherapists who treated LBP were asked to contact the researchers to help with the study. Four clinical sites were identified and set up for participation in the study. All four clinical sites were free-standing outpatient orthopaedic physiotherapy clinics. Written consent was obtained from the owners of the clinic allowing the study to take place in their clinics. The four clinics were geographically spread out in four different states (Iowa, Kansas, Texas and Utah). Each therapist was educated on the aims of the study, measurement tools used in the study, completion of forms and delivery of the intervention – EG, EEG and CG words – as well as technical delivery of the US. Education consisted of a phone call with simultaneous online video conferencing reviewing all the self-report forms as well as the physical measurements used in the study. Each therapist was deemed ready for the study when they were able to verbalise and demonstrate a clear understanding of the protocol, including measurements described below. The average session lasted 30 minutes. No inter-rate reliability was established or random data sets checked for reliability.

### Participants and Recruitment

The physiotherapists screened all new LBP patients attending physiotherapy against the inclusion and exclusion criteria for participation in the study. Inclusion criteria were adults over the age of 18, presenting at physiotherapy with a primary complaint of LBP, LBP being present for 3 months or less, fluent in English and willing to participate in the study. Exclusion criteria included medical precautions to the use of US (metal, skin lesions, pace makers), prior spine surgery and patients with leg pain only and with no LBP. Upon meeting the inclusion criteria, patients were verbally asked to participate with an explanation of the study, followed by a written consent form with the same information in written form. Upon agreeing and signing the consent, patients were randomly allocated to one of the three arms of the study. Patients were told (per IRB Informed Consent) the purpose of the study was to explore the use of a commonly used treatment (US) in physiotherapy for LBP along with the explanation of the treatment on the immediate effects of pain, forward bending and SLR. Sample size was calculated using the Repeated Measures Analysis module in PASS 14 and was based on the primary outcome measure of LBP. This analysis used a Greenhouse–Geisser Corrected *F* Test for the interaction effect among the three groups from pre- to post-assessment. In order to detect an effect size difference of 0.35 between the three groups and using a power of 70%, a total of 66 participants (22 participants in each group) would be needed. The analysis was based on a two-sided test with the significance level at 0.05.

### Intervention

All patients received the same US treatment in regards to intensity, frequency, duration and location, based on previous US for LBP studies – 1.5 Watts/cm^2^ for 10 minutes at 1 Megahertz, pulsed 20% over a 20 cm^2^ area (4 × the effective radiating area of the 5 cm US head) (Artho *et al*. 2002; Ebadi *et al*. 2013, 2014; Wong *et al*. 2007). Two parameters were different between the EG, EEG and the CG. First, the words describing the US were different:

CG: *Ultrasound has been used in the treatment of LBP. Sound waves are generated by the US machine. These sound waves result in an increased circulation in and around the injured area, including blood flow, which is needed for recovery. You might feel some warmth or heating with the treatment, which is common. We use US gel to allow the sound waves to enter your tissues and keep moving it to cover the affected area, in a figure-8 fashion.*

EG: *Ultrasound has been used in the treatment of LBP. Sound waves are generated by the US machine. These sound waves result in an increased circulation in and around the injured area, including blood flow, which is needed for recovery. You might feel some warmth or heating with the treatment, which is common. We use US gel to allow the sound waves to enter your tissues and keep moving it to cover the affected area. Typically we move the US head in a figure-8 fashion, but some new research has indicated if we perform it clockwise, it helps reduce pain even more.*

EEG: *Ultrasound has been used in the treatment of LBP to help with healing process and control pain. It utilises sound waves which help with the healing properties of tissues. Most often individuals see immediate improvements in pain and being able to bend and move further. Sometimes mild heat may be felt with the use of ultrasound but not always.*

Specifically for your back, new research has shown that by applying the ultrasound clockwise, which we are going to do today, improves the effects of the ultrasound. This improved effect helps unwind the tightness felt and eases back and leg pain a lot. The clockwise ultrasound usually will allow you to bend further forward when we test you after treatment and also you will be able to raise your leg up higher.

The second difference, because of the explanations, was in the delivery of the US in terms of skin application patterns. The CG received US covering the 20 cm^2^ in a figure-8 fashion and the EG and EEG using a clockwise circular motion.

## Outcome Measures

Prior to treatment, all study subjects completed a demographics survey capturing age, gender, ethnicity, income and duration of LBP. Additionally, subjects were asked in regards to any previous exposure to therapeutic US treatments, their perceived benefit from previous US (Likert scale), familiarity with US (Likert scale) and their belief of US’s ability to help their LBP (Likert scale) (Appendix 1). All patients additionally completed an Oswestry Disability Index to ascertain their level of disability at the time of enrolment into the study. Three measurements were taken prior to, and immediately following, US to determine the efficacy of the different treatments:

- *Pain*: LBP and leg pain were measured using Numeric Pain Rating Scale (NPRS), as has been used in various spinal pain studies (Moseley 2002, 2003, 2005). The minimal detectable change (MDC) for the NPRS is reported to be 2.1 (Cleland, Childs & Whitman 2008a).
- *Lumbar flexion*: Active trunk forward flexion, measured from the longest finger on the dominant hand to the floor (Moseley 2004; Moseley, Hodges & Nicholas 2004; Zimney, Louw & Puentedura 2014). MDC for active trunk forward flexion has been reported as 4.5 cm (Ekedahl, Jonsson & Frobell 2012).
- *Straight leg raise*: We used the SLR as a neurodynamic measurement rather than a test of hamstring length. SLR was measured with an inclinometer placed on the tibial plateau 5 cm distal to the inferior border of the patella on the most affected leg (Moseley 2004; Moseley *et al*. 2004; Zimney *et al*. 2014). MDC for SLR has been reported as a 5.7-degree difference (Ekedahl *et al*. 2012).
- Patients completed the NPRS, underwent lumbar flexion and SLR testing immediately prior to US and immediately after US. Pre- and post-treatment measurements were performed by the therapists who provided the US interventions (AL, ML, BC and JM). Following capture of the outcome measures and US, physiotherapists continued their treatment per their plan of care.

## Data Analysis

All data were analysed using SPSS version 22.0 (SPSS Inc., Chicago, IL, USA). Level of significance was set at α = 0.05. To address the aims of the trial, 2 (time: pre and post) × 3 (group: experimental (1 time), experimental (3 times) and control mixed-factorial analyses of variance were conducted for each of the dependent variables (LBP, leg pain, lumbar flexion and SLR). If an interaction was observed, then simple main effects were tested. Chi-square analyses were conducted to determine if there were differences in the proportion of participants across the three groups who improved beyond the MDC for each of the outcomes. Before analysis, all three groups showed close to normal distributions for each outcome measures considering skewness and kurtosis values within ±2.

## Results

### Patients

The randomised controlled trial comprises data from 67 patients (Table 1), with a mean age of 44.6 years and a mean duration of LBP of 6.45 weeks. Forty patients were female (59.7%). The mean NRS for LBP was 4.01 and mean Oswestry Disability Index was 27.52% indicating moderate disability. Twenty-four patients (35.8%) reported having received US in therapy before and rated US helpful as 7.04 out of a maximum score of 10. Overall patient familiarity with US was evenly spread (1 = strongly agree being familiar with US; 5 = strongly disagree being familiar with US) with a mean score of 2.54, as was their rating of their belief US would help their current LBP episode (mean 2.44). There were no differences among the three groups in familiarity with US (Kruskal–Wallis H(2) = 4.013, *p* = 0.134) or their belief that US would help their current LBP episode (Kruskal–Wallis H(2) = 1.855, *p* = 0.396).

**TABLE 1:** Demographic information.

Characteristic	Result
Age (mean; years)	44.64
Female	40 (59.7%)
Duration of LBP (mean; weeks)	6.45
Disability (Oswestry; %)	27.52
LBP (mean)	4.01
Leg pain (mean)	2.24
Flexion (mean; centimetre)	19.74
Straight leg raise (mean; degrees)	60.79
Have received US before	24 (35.8%)
Familiarity with US (0–5)	2.54
Belief US will help LBP (0–5)	2.44

*Source*: Authors’ own work

LBP, low back pain; US, ultrasound.

### Ultrasound

There was no significant time by group interaction for LBP, *F*(2,64) = 1.672, *p* = 0.196, power = 0.340 (Table 2). There was a main effect for group (*p* = 0.024) and time (*p* < 0.0001). Chi-square analysis showed that there was a statistically significant association between the three groups on the proportion of those who improved beyond the MDC for LBP (CG = 5 of 27; EG = 3 of 24; EEG = 5 of 16), χ^2^(2) = 2.181, *p* = 0.336, φ = 0.180.

**TABLE 2:** Results for low back pain, leg pain, flexion and straight leg raise.

Measurement	Before	After	Difference	Number that met MDC/%
LBP (CG)	3.93	2.87	1.06	5 (18.5)
LBP (EG)	4.69	3.5	1.19	3 (12.5)
LBP (EEG)	3.16	1.25	1.91	5 (31.3)

**LBP total**	**-**	**-**	**-**	**13 (19.4)**
Leg pain (CG)	1.41	1	0.41	0 (0)
Leg pain (EG)	3.54	2.71	0.83	2 (8.3)
Leg pain (EEG)	1.72	1	0.72	0 (0)

**Leg pain total**	**-**	**-**	**-**	**2 (2.9)**
Lumbar flexion (CG)	19.13	16.82	2.31	5 (18.5)
Lumbar flexion (EG)	21.23	17.88	3.35	10 (41.7)
Lumbar flexion (EEG)	18.53	14.56	3.97	8 (50)

**Lumbar flexion total**	**-**	**-**	**-**	**23 (34.4)**
Straight leg raise (CG)	61.93	63.81	1.88	6 (22.2)
Straight leg raise (EG)	56.33	61.46	5.13	10 (41.7)
Straight leg raise (EEG)	65.56	73.75	8.19*	10 (62.5)

**Straight leg raise total**	**-**	**-**	**-**	**26 (38.8)**

*Source*: Authors own work

* Exceeds MDC

CG, control group; EEG, extra-experimental group; EG, experimental group; LBP, low back pain; MDC, minimal detectable change.

For leg pain, there was no significant time by group interaction, *F*(2,64) = 1.473, *p* = 0.237, power = 0.303 (Table 2). However, there were statistically significant main effects for group (*p* = 0.008) and time (*p* < 0.0001). Chi-square analysis showed that there was a statistically significant association between the three groups on the proportion of those who improved beyond the MDC for leg pain (CG = 0 of 27; EG = 2 of 24; EEG = 0 of 16), χ^2^(2) = 3.694, *p* = 0.158, φ = 0.235).

For lumbar flexion, there was no significant time by group interaction, *F*(2,64) = 0.988, *p* = 0.378, power = 0.215 (Table 2). There was no main effect for group (*p* = 0.750) but there was for time (*p* < 0.0001). Chi-square analysis showed that there was a statistically significant association between the three groups on the proportion of those who improved beyond the MDC for lumbar flexion (CG = 5 of 27; EG = 10 of 24; EEG = 8 of 16), χ^2^(2) = 5.310, *p* = 0.070, φ = 0.282.

**FIGURE 2:**
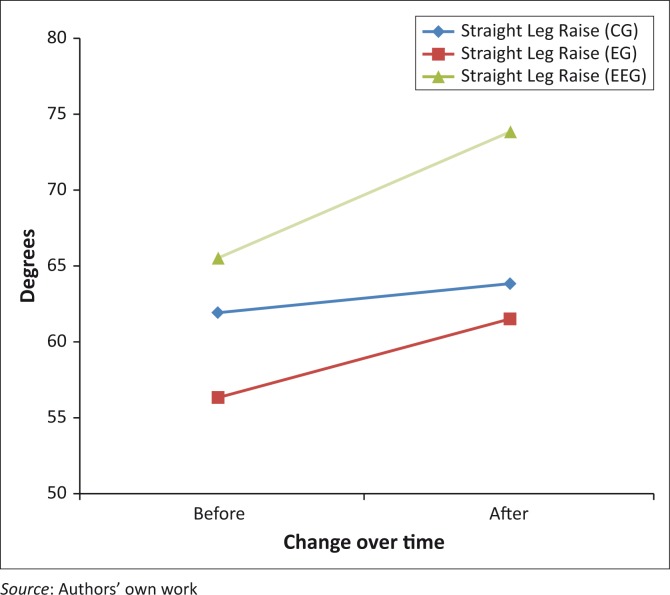
Straight leg raise before and after different ultrasound explanations.

For the SLR, there was a significant time by group interaction, *F*(2,64) = 10.469, *p* < 0.0001 (Table 2; Figure 2). Simple main effects testing using a Bonferroni correct alpha (α = 0.0125) revealed that there were no statistically significant differences at the pre-treatment (*p* = 0.307) and post-treatment (*p* = 0.138) between the two groups. Both EGs improved over time (*p* < 0.0001); however, the CG did not, *p* = 0.018. Chi-square analysis showed that there was a statistically significant association between the three groups on the proportion of those who improved beyond the MDC for SLR (CG = 6 of 27; EG = 10 of 24; EEG = 10 of 16), χ^2^(2) = 6.992, *p* = 0.030, φ = 0.323). The EEG group participants were 4.4 times (95% confidence interval: 1.1 to 17.5) more likely to improve beyond the MDC than the CG. When combining EG and EEG and comparing to the control, the experimental combined group was 3.6 times (95% confidence interval: 1.1 to 11.4) more likely to improve beyond the MDC than the CG.

## Discussion

The current study shows that using different words when applying a treatment in physiotherapy can alter the efficacy of the treatment. The results concur with previous studies that show words can ‘harm’ and words can ‘heal’ (Coppieters *et al*. 2005; Klaber-Moffett, Green & Jackson 2005; Louw, Diener & Puentedura 2014). Although the study failed to provide significant changes in LBP, leg pain and lumbar flexion, it showed that an alteration of the words used to describe therapeutic US and matching the delivery method (i.e. clockwise) has the ability to result in an immediate change in SLR, beyond MDC.

The current study shows how words influence an improvement in SLR. This concurs with recent pain neuroscience research whereby education alone has shown an immediate increase in SLR (Louw *et al*. 2015b; Louw, Diener & Puentedura 2015a; Moseley 2004; Moseley *et al*. 2004). This finding, however, is interesting given the fact that ‘only’ SLR changed, and not LBP rating, leg pain rating or flexion. Why would SLR change but not the other? The non-specific effects of treatments are complex (Benedetti 2013; Benedetti & Amanzio 2013; Bialosky *et al*. 2011) and encompass various other issues. The results of this ‘simple’ study did not aim to fully investigate this topic, but rather shed light on the importance of words chosen in delivery of treatments (Coppieters *et al*. 2005). With regards to SLR-only improvements, it could be argued that both NRS and SLR have measurement errors that surround them and the NRS is more susceptible to recall bias as the subjects were asked to recall a subjective pain rating (Childs, Piva & Fritz 2005), while in the SLR the measurement is taken by the clinician and is not susceptible to recall by the patient (Moseley 2004). It could be argued that flexion’s limited improvement (versus SLR) may be because of the well-described provocative nature of spinal flexion in patients with acute LBP (George, Fritz & Mcneil 2006), thus still fearful of flexion after the US treatment. In the SLR test, the patient is supine and the leg is raised to induce hip flexion, but in a different context – supine, unloaded and likely associated with comfort (Gifford 2014), versus a standing forward flexion movement associated with pain (George *et al*. 2006). Regardless of the exact mechanism why SLR-alone changed, the study concurs with various studies showing how choice of words impact patient outcomes. Clinicians should take note that the words they chose can enhance a treatment outcome and also calls for a much larger bio-psycho-social view of therapy in general (Coppieters *et al*. 2005; Wilson *et al*. 2009). For example, traditional orthopaedic manual therapy would imply that when a certain technique is applied to a patient and there is improvement, the improvement is only because of the technique (Bialosky *et al*. 2011). This approach would give little to no regard to the patient’s expectation, beliefs and the surrounding environment and various aspects associated with the clinician performing the technique, such as accent, experience, confidence and gender (Benedetti 2013).

A second interesting finding associated with this study is a possibility of a sub-grouping of LBP patients. Current research into LBP is strongly advocating for a sub-grouping of LBP (Fritz, Cleland & Childs 2007a). It is believed that in any representative sample of people attending physiotherapy for LBP, there is a sub-group that will respond favourably to spinal manipulation (Childs *et al*. 2004), spinal stabilisation exercises (Hicks *et al*. 2005), directional preference (Hefford 2008), traction (Fritz *et al*. 2007b). In the current study, even though SLR was the only measurement that showed a statistically significant difference between the groups, numerous patients in each group met or exceeded the MDC for LBP, leg pain and flexion (Cleland, Fritz & Brennan 2008b). For example, nearly one in five (19.4%) of the patients who received US met or exceeded the MDC of 2.1 for LBP, while more than one-third (34.4%) of patients receiving US for LBP met or exceeded the 4.5-cm MDC for spinal flexion. Current best evidence calls into question treatments such as therapeutic US given the lack of evidence (Ebadi *et al*. 2014). It could, however, once again be argued that with careful sub-grouping, there may indeed be a small group of patients who could gain significant benefit from US by improving their SLR, flexion and pain rating, albeit ‘clockwise’.

## Limitations

This study contains various limitations. Given the inclusion and exclusion criteria, that is, English, duration of LBP, extrapolating results to other populations (i.e. chronic pain) may be limited. A significant limitation is that the US (apart from words) were performed differently – figure-8 versus clockwise and it is assume this has little effect on the therapeutic outcome, apart from matching the words of the US instruction. An added limitation is the fact that clinical sites were recruited from a newsletter associated with a seminar group with a bias towards a pain science approach, which may have impacted the delivery of the education. Although care was taken to train the therapists in the collection of the data, using standardised tests and well-described protocols, inter-rater reliability was not specifically assessed for this study. The fact that there were noticeable differences in the number of patients allocated to each of the three arms of the study resulted in an uneven distribution, which likely impacted the results, biasing towards a specific group.

## Conclusion

Using different words when applying a treatment in physiotherapy can alter the efficacy of the treatment. The results from this study concur with previous studies that show words can ‘harm’ and words can ‘heal’. Results showed that an alteration of the words used to describe therapeutic US and matching the delivery method (i.e. clockwise) has the ability to result in an immediate change in SLR, beyond MDC. Future studies should examine if there is a subgroup of patients who may benefit from US and additionally explore various TA issues that may impact its efficacy.

## Appendix 1: Additional questions regarding ultrasound

1. **Have you ever received ultrasound therapy in the past for any previous injuries?**
  □ No
  □ Yes: low back pain
  □ Yes: other body area: _________
  If yes – you HAVE received ultrasound therapy before: How helpful do you think it was?
2. **I am familiar with how ultrasound therapy works and the benefits of it?**
  □ Strongly agree
  □ Agree
  □ Undecided
  □ Disagree
  □ Strongly disagree
3. **Ultrasound therapy will help my back pain?**
  □ Strongly agree
  □ Agree
  □ Undecided
  □ Disagree
  □ Strongly disagree
